# The fudan tinnitus relieving system application for tinnitus management

**DOI:** 10.1186/s12911-023-02164-w

**Published:** 2023-04-21

**Authors:** Dongmei Tang, Haiyan Wang, Dantong Gu, Lei Ye, Shan Sun, Huawei Li

**Affiliations:** 1 ENT institute and Department of Otorhinolaryngology, Eye & ENT Hospital, State Key Laboratory of Medical Neurobiology and MOE Frontiers Center for Brain Science, Fudan University, 83 Fenyang Road, Shanghai, 200031 PR China; 2 Institutes of Biomedical Sciences, Fudan University, 138 Yixueyuan Road, Shanghai, 200032 PR China; 3 NHC Key Laboratory of Hearing Medicine, Fudan University, 83 Fenyang Road, Shanghai, 200031 PR China; 4 Shanghai ZEHNIT Medical Technology Co., Ltd, 908 Ziping Road, Shanghai, 201203 PR China; 5 Clinical Research Unit of Eye & ENT Hospital, Fudan University, 83 Fenyang Road, Shanghai, 200031 PR China; 6 The Institutes of Brain Science and the Collaborative Innovation Center for Brain Science, Fudan University, 138 Yixueyuan Road, Shanghai, 200032 PR China

**Keywords:** Fudan Tinnitus Relieving System (FTRS), Tinnitus management, Mobile app, Sound therapy

## Abstract

**Objective:**

Tinnitus is a highly prevalent hearing disorder, and the burden of tinnitus diagnosis and treatment is very heavy, especially in China. In order to better benefit the majority of tinnitus patients, we developed a new mobile app based on our patented invention – named the Fudan Tinnitus Relieving System (FTRS) – for tinnitus management. The FTRS app aims to alleviate patients’ tinnitus symptoms using customized sound therapy, to evaluate the treatment effect, to provide a doctor-patient communication platform, and to support tinnitus rehabilitation and auditory health.

**Methods:**

In this study, we introduced the major functions of the FTRS app, analyzed the geographical distribution of users around China, and performed an analysis on the demographic and clinical characteristics of patients with tinnitus, including age and tinnitus position, duration, frequency, and severity in both men and women based on the user information collected by the FTRS. The data for 22,867 participants (males: 13,715; females: 9,152) were included in the statistical analysis.

**Results:**

The FTRS app has been popular with tinnitus patients since its launch in May 2018 with its integrated pitch-matching test, individualized sound therapy, follow-up assessment, and provision of easy-to-understand science and education for tinnitus. The users were located throughout Mainland China but primarily concentrated in Shanghai, Jiangsu, Zhejiang, Guangdong, and Shandong provinces. We observed gender differences regarding age and tinnitus frequency, severity, and position among the app’s users. The FTRS has not only facilitated patients’ access to treatment at times and places that are convenient for them, but also provides a large amount of data based on user feedback in order to support clinical tinnitus research.

**Conclusions:**

Compared with traditional face-to-face medical treatment, the FTRS greatly reduced medical costs and enabled patients with tinnitus to arrange their own treatment times. At the same time, the FTRS has provided standardized tinnitus data that have laid a foundation for clinical research on tinnitus. However, because of differences in the popularity and utilization of smart devices, FTRS user data might only reflect the situation of tinnitus patients who can effectively use smart devices. Therefore, the findings of this study need to be interpreted with caution.

## Introduction

Tinnitus, the phantom perception of sound without a corresponding external stimulus, is a common disorder that causes significant morbidity. The severity of tinnitus varies, and a large percentage of patients only experience minimal impairment during their daily routines, while severe cases of tinnitus can involve anxiety, depression, insomnia, and concentration problems that can significantly impair quality of life [1]. Epidemiological studies of tinnitus indicate a prevalence of 6–26% with 1.2–1.6% of patients reporting severely annoying tinnitus [2, 3]. There are currently no treatments that can reliably reduce the perception of tinnitus.

Sound therapy is a widely applied method for treating tinnitus in the clinic. However, there are many challenges that need to be addressed, such as the long duration of sound therapy, which affects patient adherence; limited professional guidance and communication; low compliance to acoustic therapy; and the tremendous differences in efficacy among individuals. In recent years, smartphones, smartphone apps, and auxiliary health devices such as heart monitors and smart wristbands have garnered considerable popularity in helping patients to monitor and treat health problems [4–6]. Smart mobile applications have also made significant strides in tinnitus management. Specifically, the use of mobile apps to deliver tinnitus management provides several advantages, including the following: (1) mobile smart devices offer a well-established ecosystem that is easily discoverable and accessible via various mobile platforms, thereby increasing access to health care [7]; (2) medical professionals can guide and monitor patients’ tinnitus treatment remotely, thus making tinnitus management more convenient and thereby improving the quality of medical services [8]; (3) patients can freely choose the times to use portable electronic devices thus making treatment more convenient; and (4) these tinnitus management solutions can be designed or customized directly on the mobile device to aid in reducing patients’ tinnitus symptoms, which can reduce the cost of tinnitus management [9]. Nevertheless, there are also potential issues associated with mobile app use, and these include screened and proper use of the app [10]. Although numerous health management applications are currently available, it is not known whether the recommendations and interventions in the health management programs conform to the latest evidence from clinical practice. In addition, users lack professional knowledge to identify and choose accurate and appropriate treatment methods. Different apps are designed with different priorities, and even apps that purport to complete the same task often include extra functionality or features, making it difficult to effectively assess and compare various apps. Furthermore, regarding responsibility and risk [11], the ethical and security aspects of data processing need to be considered when accessing user data via mobile devices. In addition, applications that directly affect clinical decision-making (including drug dose calculation and treatment plan adjustment) need to be implemented more carefully. Here, we present a new tinnitus management app that adopts customized sound therapy as the treatment method, named the Fudan Tinnitus Relieving System (FTRS). The FTRS is available for iOS and Android systems, and patients can complete an entire set of acoustic therapy processes online for free. Here, we provide an overview of the FTRS platform and discuss the correlation between gender and tinnitus characteristics based on user retention data.

## Methods

### Participants

**Fig. 1:**
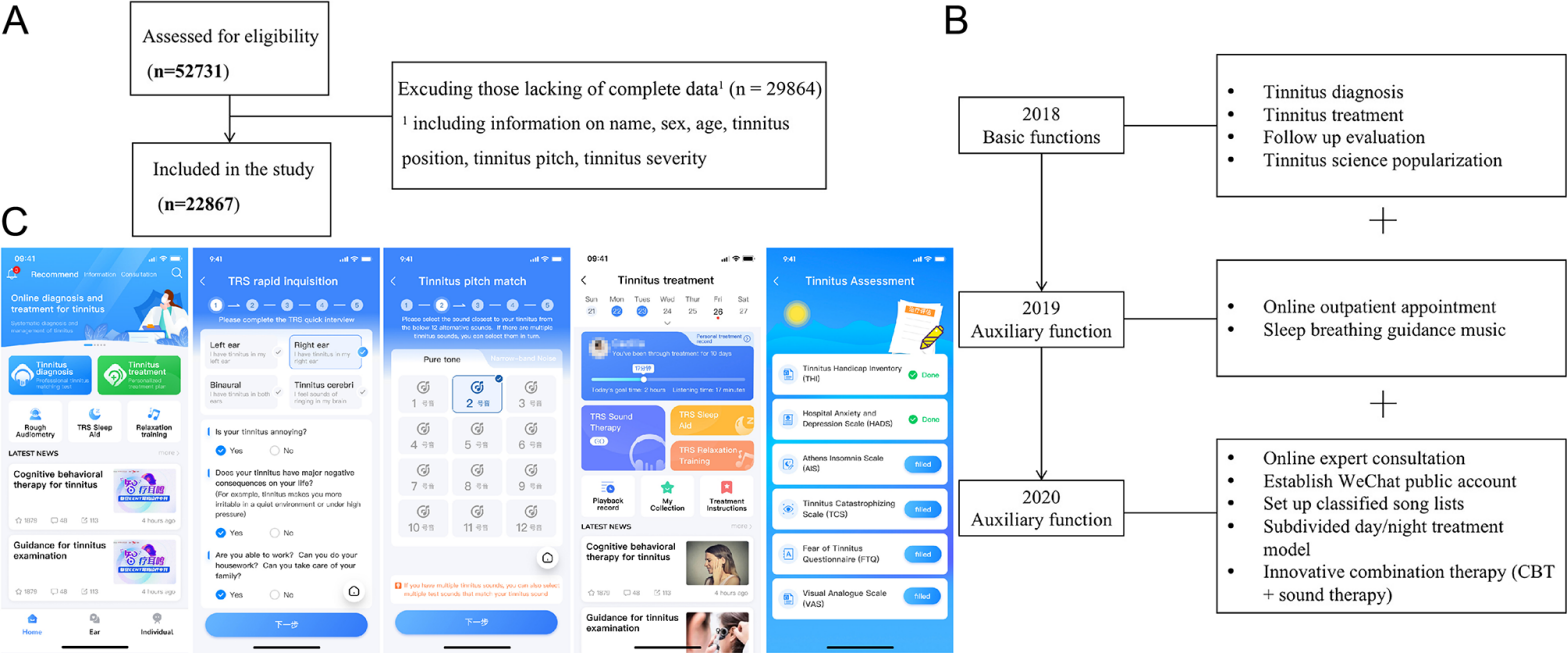
Data screening procedure and the introduction of the FTRS application

The total number of FTRS app users reached 52,731 by 2020. Excluding users with missing information, the data from 22,867 participants were collected (Fig. 1A). Using the information retained from users, we retrospectively analyzed the correlation between gender and tinnitus characteristics (Table 1).

**Table 1:** Descriptive data of FTRS App users

	Male(n = 13,715)	Female(n = 9152)	*p*
**Age in years (median (IQR))**	34.00 (27.00, 43.00)	36.00 (28.00, 48.00)	< 0.001
**Count of tinnitus sound types (n, %)**			< 0.001
**1 sound**	11,306 (82.4)	7094 (77.5)	
**2 sounds**	2004 (14.6)	1682 (18.4)	
**3 sounds**	306 (2.2)	279 (3.0)	
**4 sounds**	99 (0.7)	93 (1.0)	
**5 sounds**	0 (0.0)	4 (0.1)	
**Dominant matched pitch(Hz)(n, %)**			< 0.001
**125**	1095 (8.0)	1563 (17.1)	
**250**	722 (5.3)	1000 (10.9)	
**500**	437 (3.2)	541 (5.9)	
**1000**	520 (3.8)	479 (5.2)	
**2000**	509 (3.7)	357 (3.9)	
**3000**	473 (3.4)	260 (2.8)	
**4000**	720 (5.2)	303 (3.3)	
**5000**	881 (6.4)	419 (4.6)	
**6000**	1312 (9.6)	606 (6.6)	
**8000**	1755 (12.8)	897 (9.8)	
**10,000**	2149 (15.7)	1074 (11.7)	
**12,000**	3142 (22.9)	1653 (18.1)	
**Tinnitus position (n, %)**			< 0.001
**Tinnitus cerebri**	1189 (8.7)	727 (7.9)	
**Both ears**	5968 (43.5)	3451 (37.7)	
**Right ear**	2964 (21.6)	2326 (25.4)	
**Left ear**	3594 (26.2)	2648 (28.9)	
**Tinnitus severity (n, %)**			< 0.001
**1**	1466 (10.7)	990 (10.8)	
**2**	4036 (29.4)	2351 (25.7)	
**3**	3411 (24.9)	2530 (27.6)	
**4**	4802 (35.0)	3281 (35.9)	
**Tinnitus duration (months) (median (IQR))**	9.00 (2.00, 36.00)	6.00 (2.00, 24.00)	< 0.001

### Materials

Figure 1B presents the development of the FTRS app.

### Measurements

Based on existing clinical acoustic therapy, the FTRS includes separate modules for “tinnitus diagnosis”, “tinnitus treatment”, “follow-up evaluation”, and “tinnitus science popularization”. Screenshots of the diagnosis, treatment, and evaluation using the FTRS app are shown in Fig. 1C. The steps in using the app were as follows:

*User registration*. The user downloads the FTRS app through the iOS or Android system and completes the registration according to the prompts in the app.

*Tinnitus diagnosis*. The tinnitus diagnosis requires users to undergo a test using headphones in a relatively quiet environment. Clinical studies have demonstrated that customized sound therapy based on personal hearing and tinnitus is more effective than non-customized sound therapy [12, 13]. The tinnitus diagnosis includes fast pitch matching and fine pitch matching to detect the frequency and loudness of the tinnitus. If users want to test their hearing, they can choose the acoustic test to obtain their approximate air conduction hearing threshold.

*Tinnitus treatment*. The tinnitus frequency and loudness are obtained according to the tinnitus diagnosis, and customized treatment music is generated. Because patients have different preferences for types of sound therapy, various types of sound therapy song lists are listed in the FTRS, and this enables patients to choose the most comfortable and relaxing stimulation sound for treatment. It is recommended that users adhere to the treatment for more than 2 h a day, and the app will provide reminders to recheck and test the severity of the tinnitus every 3 months after the initiation of sound therapy.

*Follow-up evaluation*. Studies have shown that the frequency and loudness of tinnitus may change over the course of treatment. In order to be aware of the severity of their tinnitus and the effect of acoustic treatment in a timely manner, users need to be followed up and perform regular evaluations. The first evaluation is performed before the start of treatment, and the patient is followed up every 3 months. Evaluation is conducted in the form of a questionnaire consisting of the following six components: the Tinnitus Handicap Inventory (THI) [14], the Hospital Anxiety and Depression Scale (HADS) [15], the Athens Insomnia Scale (AIS) [16], a visual analog scale (VAS) [17], the Fear of Tinnitus Questionnaire (FTQ) [18], and the Tinnitus Catastrophizing Scale (TCS) [19]. Users can obtain their follow up history in the application.

*Tinnitus science popularization*. Some researchers have found that the intervention effect of acoustic stimulation itself on tinnitus is unclear and have proposed that the combination of acoustic stimulation and tinnitus education will be more effective in improving tinnitus [20, 21]. The FTRS includes the “Tinnitus Science Popularization” module to disseminate popular-science knowledge related to tinnitus in order to assist patients in correctly understanding tinnitus and in helping them keep a positive attitude when treating their tinnitus.

### Statistical analysis

Demographics and baseline characteristics such as age, dominant tinnitus-matched frequency, tinnitus position, and severity of tinnitus, were categorized by gender. The relationships between tinnitus duration and tinnitus characteristics were also analyzed. We summarized the descriptive statistics of continuous variables as medians and interquartile intervals (IQRs) and summarized the number of patients as well as the proportion of patients at each level of the categorical variables. Differences in characteristics between the groups were measured with chi-square/adjusted chi-square tests for categorical variables and with Wilcoxon tests for continuous variables. We created a geographic information system map to show the locations of the patients, and box-plots, density plots, bar plots, and mosaic plots were used to highlight the distributions of patients with different characteristics. Geographical information of China’s provinces, including the mainland, Taiwan, Hong Kong, and the South China Sea Islands have been organized into shp/dbf/shx formats, which are freely accessible for online query and download. We got the shp database of China’s GIS information from the National Catalogue Service For Geographic Information (www.webmap.cn). The panel we showed in the article added patients’ province information from our clinical database. The gis map was plotted by R software (version 4.0.5) using “magrittr”, “ hchinamap”, “rgdal”, “sp”, and “mapproj”. All statistical analyses were performed using R software (version 4.0.5, RStudio, Inc.), and two-sided *p*-values < 0.05 were considered statistically significant.

## Results

### Regional differences in user distribution

**Fig. 2:**
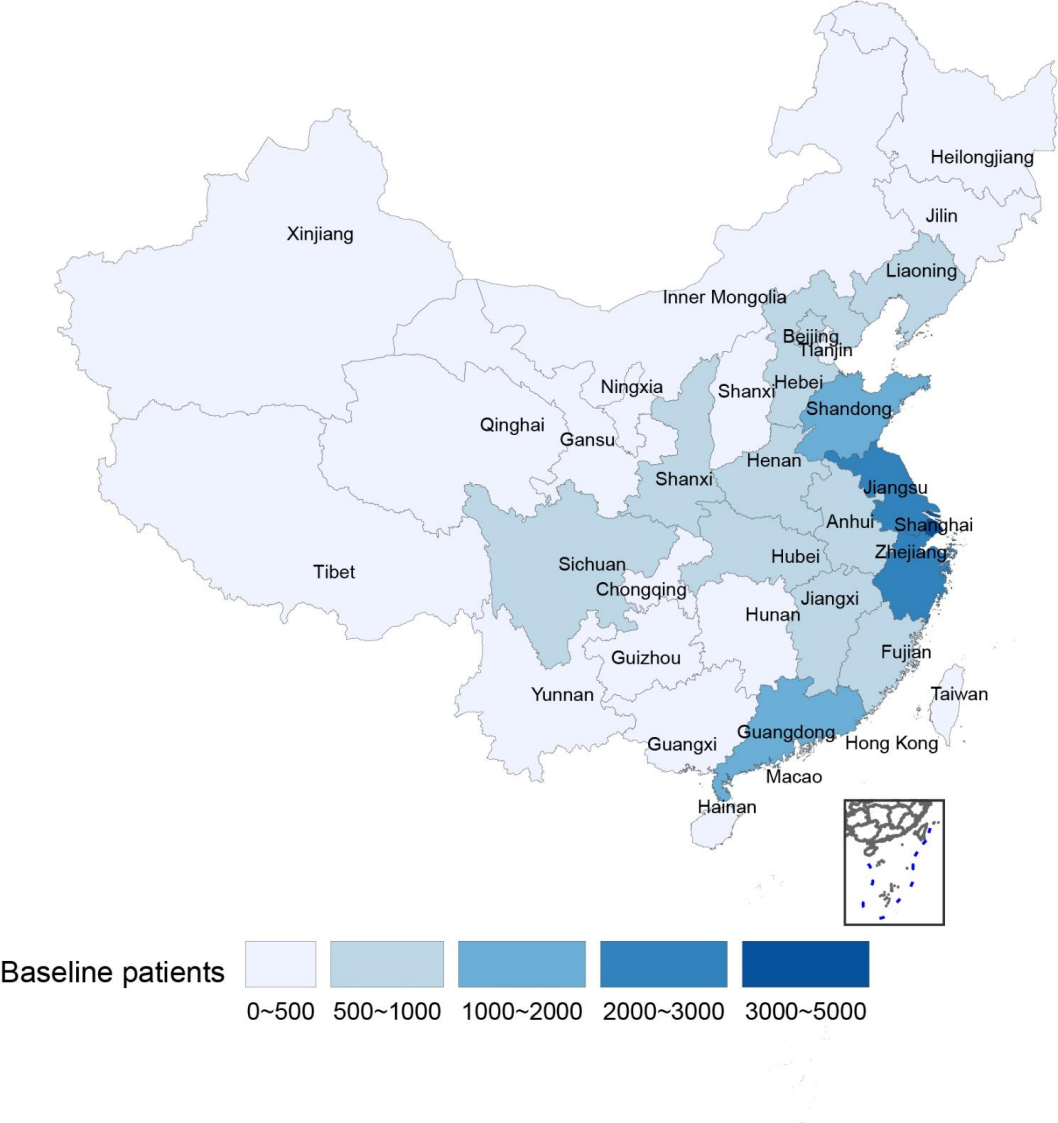
Geographical distribution of FTRS app users in China Users of the FTRS app are unevenly distributed in different provinces of China. The light to dark color block represents different quantitative grades of FTRS users, i.e., 0–500, 500–1000, 1000–2000, 2000–3000, and 3000–5000

We used the participants’ registered mobile phone numbers to trace their geographical location, and the results are shown in Fig. 2. There were large regional differences in the distribution of patients using the FTRS. Among them, Jiangsu, Zhejiang, and Shanghai had the most users, with 3000–5000 users in Shanghai and 2000–3000 users in Zhejiang and Jiangsu provinces, respectively. Shandong and Guangdong both had 1000–2000 users. In contrast, northwest China had the fewest users, ranging from dozens to a few hundred in the various provinces (Table 2).

**Table 2:** Geographical distribution of FTRS App users

Province	Number of users
Beijing	884
Tianjin	275
Hebei	611
Shanxi	352
Inner Mongolia	157
Liaoning	543
Jilin	219
Heilongjiang	230
Shanghai	4562
Jiangsu	2730
Zhejiang	2319
Anhui	980
Fujian	686
Jiangxi	769
Shandong	1094
Henan	902
Hubei	615
Hunan	451
Guangdong	1461
Guangxi	215
Hainan	43
Chongqing	317
Sichuan	747
Guizhou	232
Yunnan	338
Tibet	50
Shanxi	623
Gansu	198
Qinghai	83
Ningxia	46
Xinjiang	201
Taiwan	0
Hong Kong	0
Macau	0
South China Sea Islands	0

### Age and gender differences of the users

**Fig. 3:**
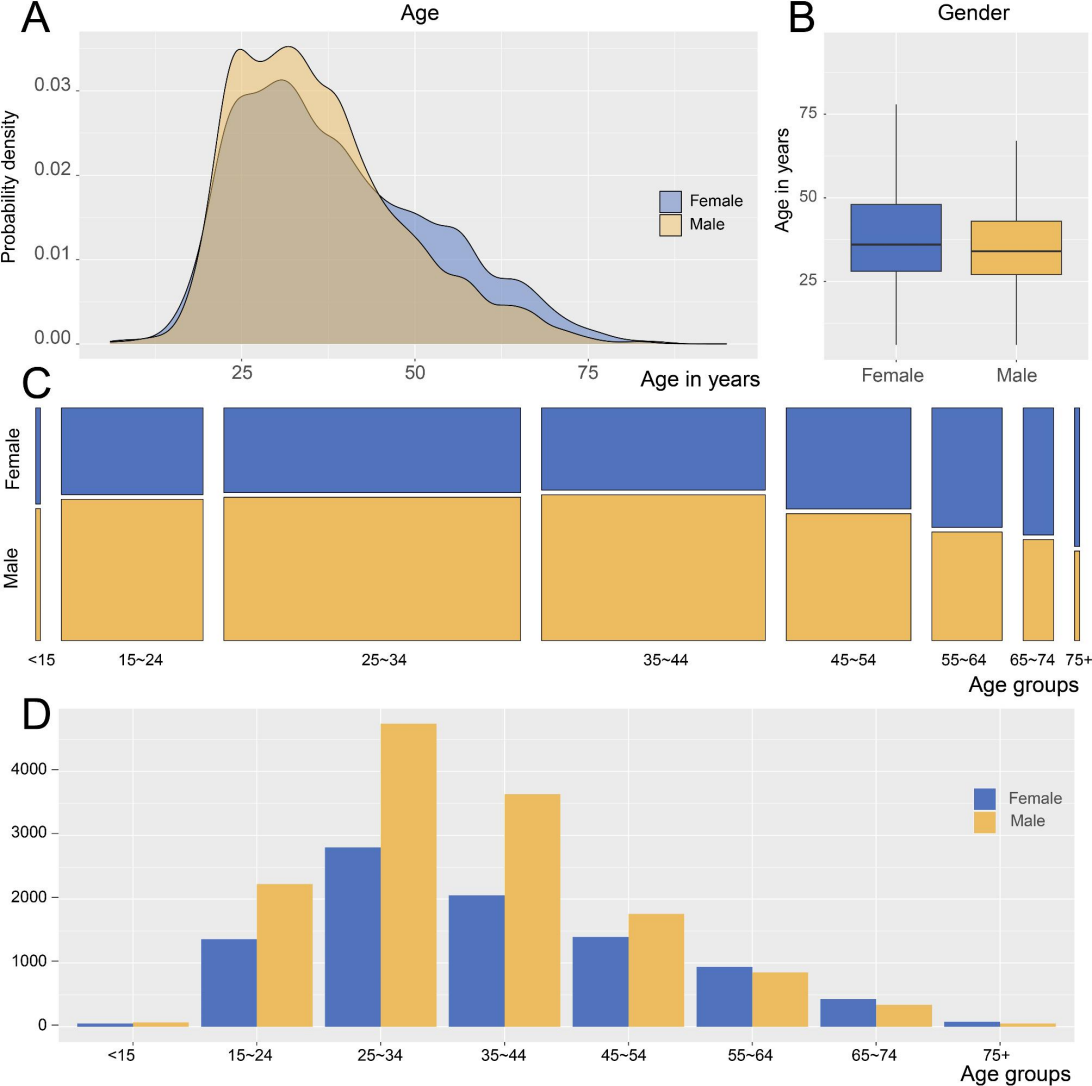
Age distribution of FTRS app users according to gender (**A**) Probability density of every age among male and female tinnitus patients. (**B**) Median age of male and female users. Data are shown as medians (IQR), and the upper edge represents the largest value and the lower edge represents the smallest value. (**C**) Gender differences of tinnitus patients in different age groups. Each corresponding area represents the number of users in the age group. (**D**) Number of users in different age groups and genders. Yellow indicates data for men, blue indicates data for women, and brown in (**A**) indicates the overlapping region of both genders

There were significantly more male users (13,715 users) compared to female users (9,152 users) (Table 1). In general, most app users were in the 25–44-year-old age group followed by those in the 15–24, 45–54, and 55–64-year-old age groups, with very few users over 65 years old (Fig. 3A-D). In this study, the average ages of male and female users were 34 years (IQR 27.00, 43.00) and 36 years (IQR 28.00, 48.00), respectively (Fig. 3B). Interestingly, in the 15–44-year age group the proportion of male app users was significantly higher than that of female users (Fig. 3A and C), whereas the proportion of female users gradually increased after the age of 45 years, presenting two significant probability density peaks for different age groups (Fig. 3A) and a decreasing curve for the gender ratio after the age of 45 (Fig. 3C).

### Gender-tinnitus frequency differences

**Fig. 4:**
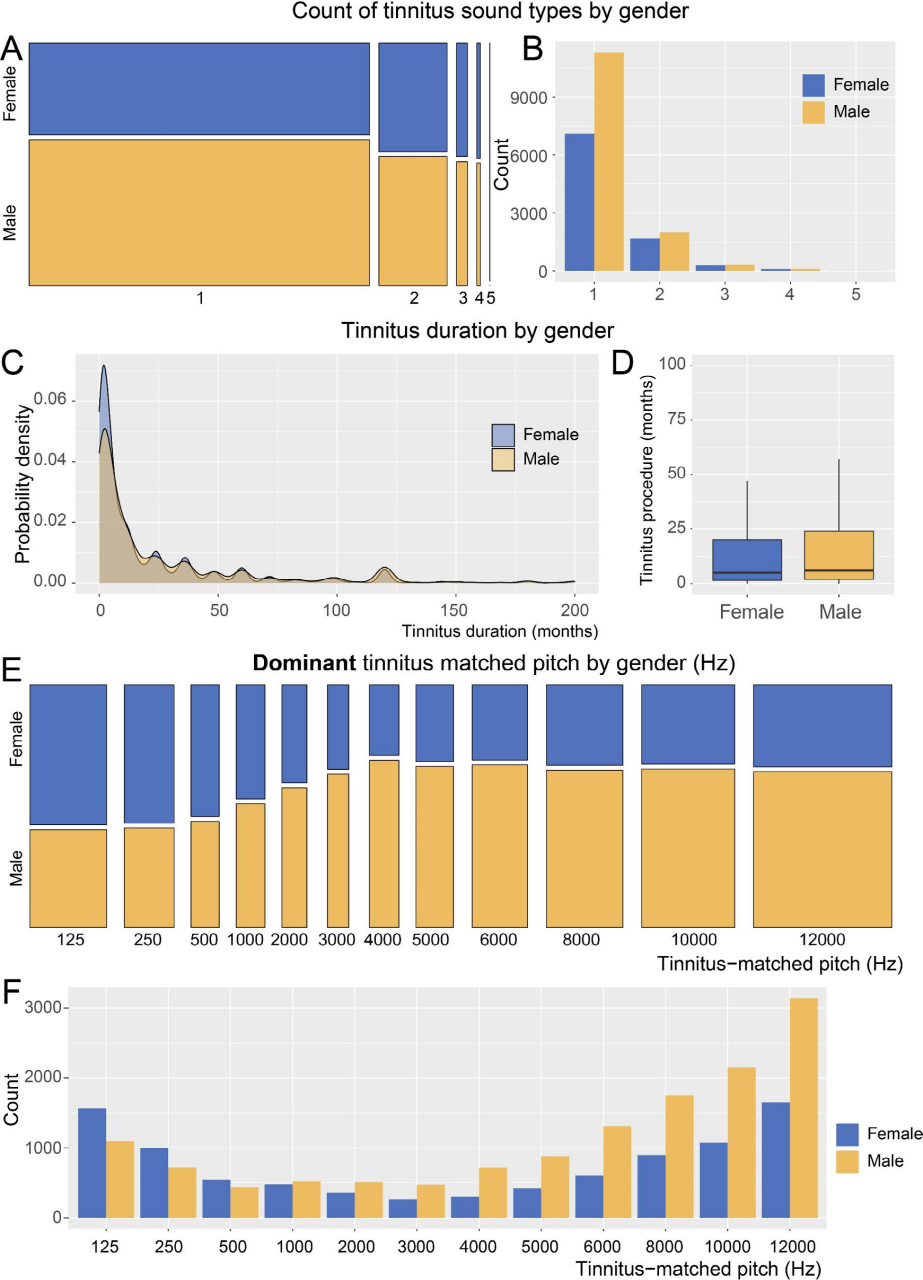
Distribution of tinnitus frequency according to gender (**A**) Proportion of 1 to 5 sound types of tinnitus for users by gender. The area of each mosaic represented the proportion of corresponding tinnitus users. (**B**) The numbers of the frequency components of tinnitus by gender. (**C**) Probability density of tinnitus duration among male and female tinnitus patients. (**D**) The average tinnitus duration (months) of male and female users. (**E**) Proportions of male and female users with different dominant tinnitus-matched pitches. The area of each mosaic represented the proportion of corresponding tinnitus users. (**F**) Count of male and female users with different tinnitus-matched pitches. Yellow indicates the data for men, blue indicates the data for women, and brown in (**C**) indicates the overlapping region of both genders

Individuals with tinnitus exhibited various types of tinnitus, such as the sound of cicadas, ringing, hissing, squeaking, and buzzing, and some patients reported that their tinnitus had many components. In order to understand the tinnitus types vividly and concretely, we used the app to analyze the types of tinnitus. The results were shown in Fig. 4; Table 1. Most FTRS users suffered from a single type of tinnitus, a few users reported 2–3 types of tinnitus, and a very few users had complex tinnitus types and perceived 4–5 different sounds (Fig. 4A-B). The number of male users with only one type of tinnitus sound was significantly higher than that of female users, whereas the proportion of male and female users with two or more complex tinnitus sounds was similar (Fig. 4B, and Table 1). According to the use records of FTRS, the probability of women receiving attention and seeking treatment measures at the initial stage of tinnitus was slightly higher than that of men (Fig. 4C). The average tinnitus durations of female and male users were 6 months (IQR: 2.00, 36.00) and 9 months (IQR: 2.00, 24.00), respectively (Fig. 4D, and Table 1).

Tinnitus sound showed a significant heterogeneity. Not only did different patients hear different tones of tinnitus sounds, but the frequency also differed. We further analyzed the gender-frequency differences in the tinnitus patients and observed that among users with a dominant tinnitus frequency below 1 kHz, the proportion of female users significantly exceeded that of male users. When the dominant tinnitus frequency was above 1 kHz, the proportion of male users gradually increased and became significantly higher than that of female users. The gender-frequency difference curves exhibited a peak at 125–250 Hz (with a higher proportion of female users) and a peak at 4000 Hz (with a higher proportion of male users) (Fig. 4E, and Table 1). Among all users, mid-frequency tinnitus (1000–3000 Hz) was the rarest (Fig. 4E-F). The incidence of low-frequency tinnitus (125–500 Hz) among male users was slightly greater than that for medium frequency, but most exhibited high-frequency (4000–8000 Hz) and ultra-high frequency (> 8000 Hz) tinnitus (Fig. 4E-F). The tinnitus frequency band distribution curve for male users decreased slowly and then increased sharply from low frequency to high frequency (Fig. 4F; Table 1). The incidence of medium-frequency tinnitus was the lowest in female users, as observed in male users, but unlike male users, female users displayed similar proportions of low, high, and ultra-high-frequency tinnitus. The distribution curve of the tinnitus frequency band for female users was “U” shaped and was low in the middle and high on both ends (Fig. 4F; Table 1). In conclusion, in terms of quantity and proportion, female users accounted for the majority of dominant low-frequency tinnitus, whereas male users were dominant in the high-frequency and ultra-high-frequency tinnitus groups (Fig. 4E-F). Thus, we conclude that there are prominent gender-frequency differences among patients with tinnitus.

### Tinnitus location analysis

**Fig. 5:**
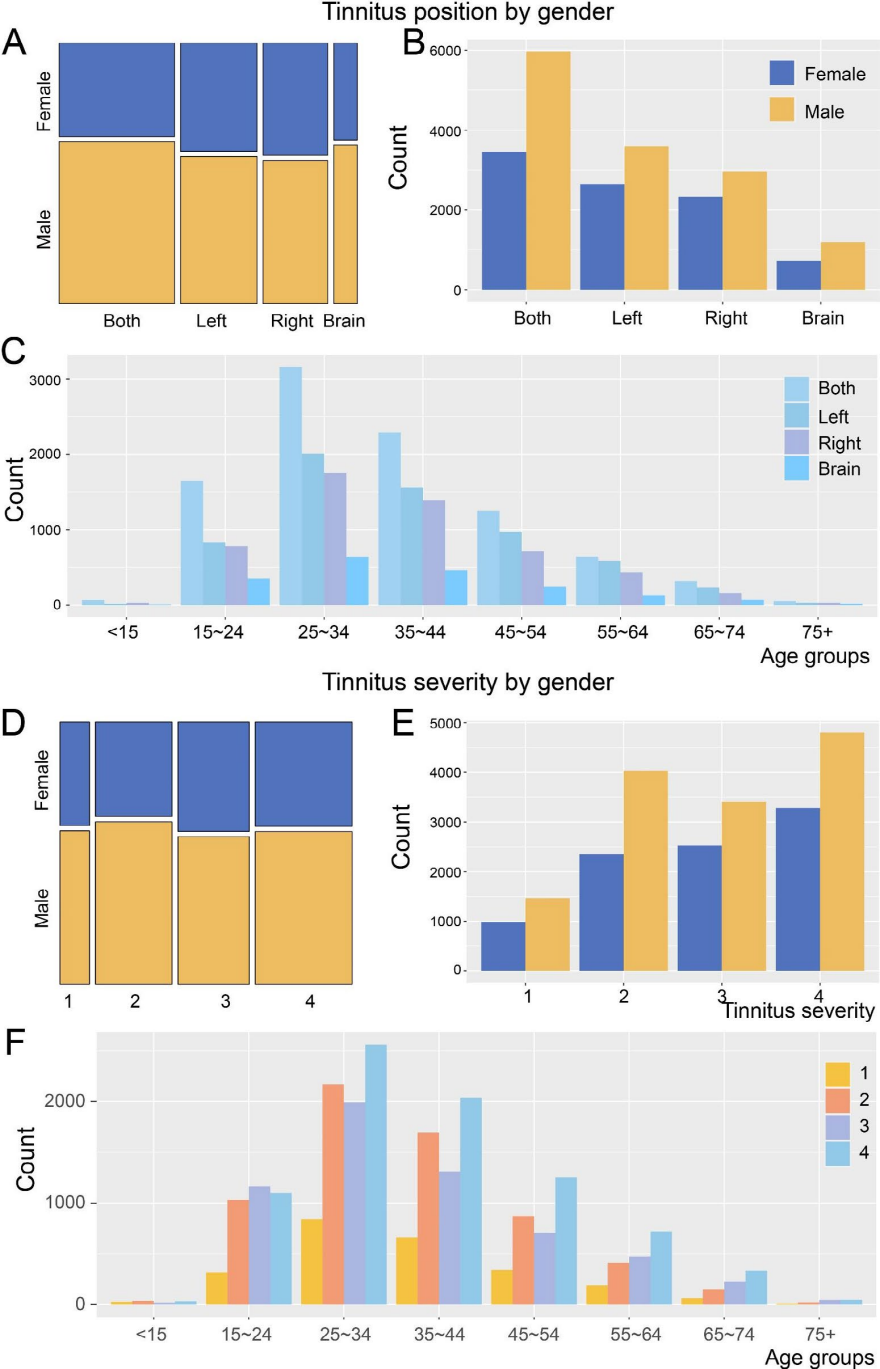
Distribution of tinnitus position and tinnitus severity according to gender (**A**) Distribution of tinnitus locations in users of different genders. The area represented the proportion of corresponding tinnitus users. (**B**) Statistics regarding the number of male and female users with different tinnitus locations. (**C**) Count of users with different locations at different ages. (**D**) Proportion of grades of tinnitus severity according to gender. Grades 1–4 represented different degrees of tinnitus severity. (**E**) Number of female users (blue bars) and male users (yellow bars) for different grades of tinnitus severity. (**F**) Distribution of users with different grades of tinnitus severity at all ages

Tinnitus can occur in a single ear (left or right), both ears, or intracranially [22]. In order to understand the tinnitus location distribution of app users, we further analyzed the gender-location relationship of tinnitus users. Among all the patients, the number of users with single ear tinnitus (including left and right ears) was the largest, followed by binaural tinnitus, while the occurrence of tinnitus cerebri was the lowest (Fig. 5A-B). In unilateral tinnitus, the probability of left tinnitus was slightly higher than that of right tinnitus. Regardless of the location of tinnitus, there was a significantly higher proportion of male than female users (Fig. 5A-B). Further analysis of tinnitus location at different ages showed similar trends with that of different genders (Fig. 5C).

### Statistics on tinnitus severity

According to the impact of tinnitus on users, the severity of tinnitus was divided into the following four levels [23]: grade 1 – No impairment, patients are not bothered by their tinnitus; grade 2 – Slight impairment, patients suffer from tinnitus under specific conditions (such as quiet environments or high stress; grade 3 – Moderate impairment, patients are permanently annoyed by their tinnitus; and grade 4 – Severe impairment, patients are heavily disturbed by their tinnitus and cannot live and work normally. According to the evaluation score from the user questionnaire, the severity of most tinnitus patients was grade 2 or above, indicating that tinnitus had varying degrees of negative impact on most users (Fig. 5D-E). Among them, the number and proportion of users with grade-4 tinnitus were the largest (Fig. 5D), which might be related to the stronger demand for tinnitus treatment among patients with severe tinnitus. The number of female users increased gradually from grade 1 to grade 4, while the tinnitus severity of male users was mainly concentrated in grade 2 and grade 4 (Fig. 5E). We further analyzed the severity of tinnitus in different age groups and found that except for patients ≤ 15 years old the severity of tinnitus at all ages was more concentrated in grades 2–4 (Fig. 5F).

### The relationship between tinnitus duration and other tinnitus characteristics

**Fig. 6:**
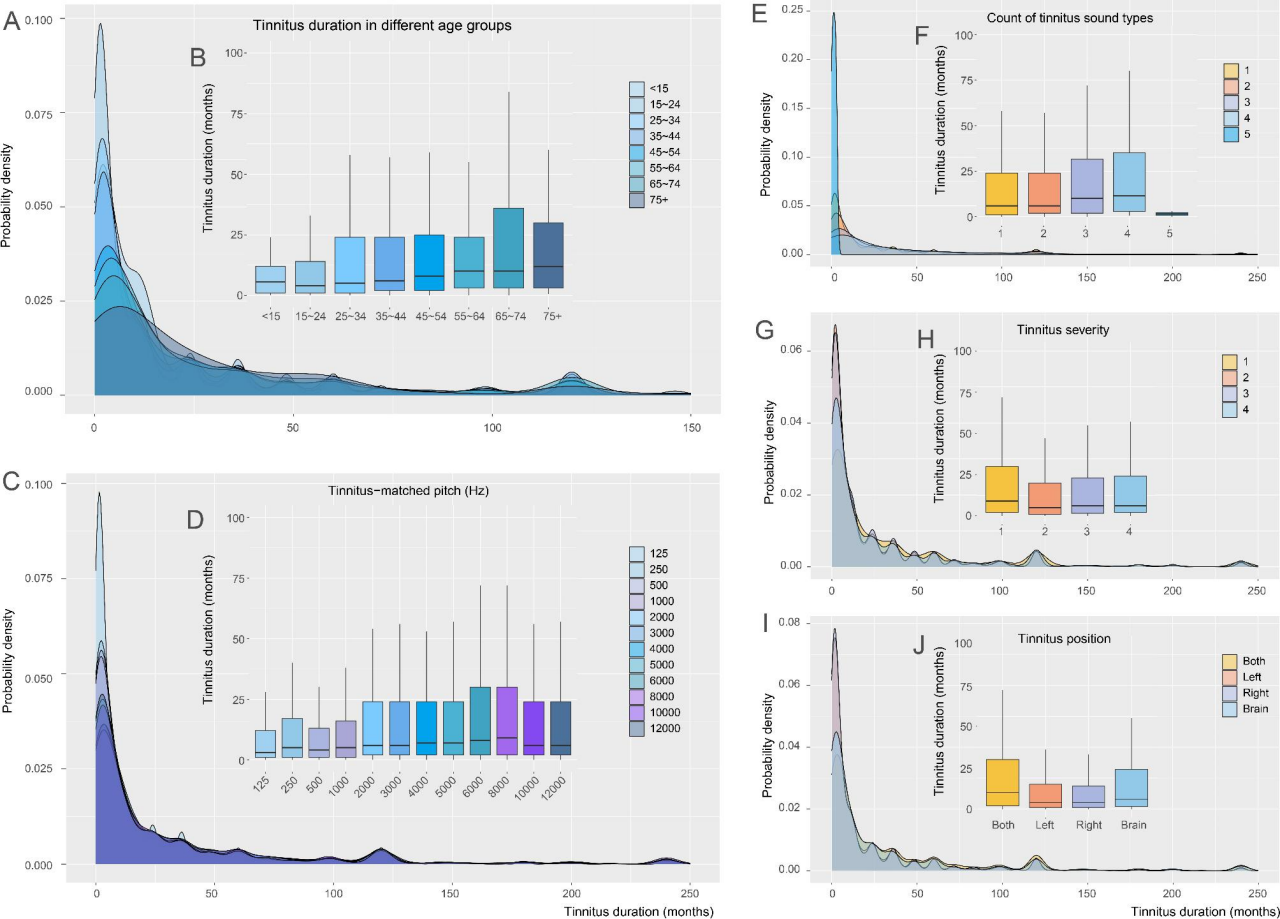
Tinnitus duration of FTRS app users according to other tinnitus characteristics (**A**) Probability density of tinnitus duration among users at all ages. (**B**) Median tinnitus duration for users at all ages. (**C**) Probability density of tinnitus duration among users with different dominant tinnitus-matched frequencies. (**D**) Median tinnitus duration for users with different dominant tinnitus-matched frequencies. (**E**) Probability density of tinnitus duration among users with different numbers of tinnitus sound types. (**F**) Median tinnitus duration for users with different numbers of tinnitus sound types. (**G**) Probability density of tinnitus duration among users with different grades of tinnitus severity. (**H**) Median tinnitus duration for users with different grades of tinnitus severity. (**I**) Probability density of tinnitus duration among users with different tinnitus locations. (**J**) Median tinnitus duration for users with different tinnitus locations

Analysis of the relationship between tinnitus duration and tinnitus characteristics suggested a significant effect of sound type, dominant matched pitch, tinnitus location, and tinnitus severity on the duration of tinnitus (Fig. 6, and Table 3). The tinnitus duration at all ages was generally below 25 months (Fig. 6A-B). Compared with patients with high-pitched tinnitus, those with low-pitched tinnitus tended to seek treatment measures relatively early and thus had a shorter course of tinnitus (Fig. 6C-D). Except for those reporting five tinnitus sound types, the tinnitus duration increased with users reporting more tinnitus sound types (Fig. 6E-F). Users grouped into grade 1 severity seemed to have the longest tinnitus duration compared to those with higher severity grades (grade 2–4) (Fig. 6G-H). Users with tinnitus perceived on a single side had shorter tinnitus duration compared with those with tinnitus in both ears or in the brain (Fig. 6I-J).

**Table 3:** Tinnitus duration according to other tinnitus characteristics

	n (%)	Tinnitus duration (months) (median (IQR))	*p*
**Count of tinnitus sound types (n, %)**			< 0.001
**1 sound**	18,400 (80.47)	7.00 [2.00, 30.00]	
**2 sounds**	3686 (16.12)	8.00 [2.00, 36.00]	
**3 sounds**	585 (2.56)	12.00 [3.00, 48.00]	
**4 sounds**	192 (0.84)	12.00 [3.00, 48.00]	
**5 sounds**	4 (0.02)	1.50 [0.75, 2.25]	
**Dominant matched pitch(Hz)(n, %)**			< 0.001
**125**	2658 (11.62)	3.00 [1.00, 12.75]	
**250**	1722 (7.53)	6.00 [1.00, 24.00]	
**500**	978 (4.28)	5.00 [1.00, 24.00]	
**1000**	999 (4.37)	6.00 [1.00, 24.00]	
**2000**	866 (3.79)	6.50 [2.00, 30.75]	
**3000**	733 (3.21)	9.00 [2.00, 36.00]	
**4000**	1023 (4.47)	10.00 [2.00, 36.00]	
**5000**	1300 (5.69)	9.00 [2.00, 36.00]	
**6000**	1918 (8.39)	12.00 [2.00, 40.00]	
**8000**	2652 (11.60)	12.00 [2.50, 48.00]	
**10,000**	3223 (14.09)	9.00 [2.00, 36.00]	
**12,000**	4795 (20.97)	9.00 [2.00, 36.00]	
**Tinnitus position (n, %)**			< 0.001
**Tinnitus cerebri**	1916 (8.38)	8.00 [2.00, 36.00]	
**Both ears**	9419 (41.19)	12.00 [3.00, 48.00]	
**Right ear**	5290 (23.13)	5.00 [1.00, 24.00]	
**Left ear**	6242 (27.30)	5.00 [1.00, 24.00]	
**Tinnitus severity (n, %)**			< 0.001
**1**	2456 (10.74)	12.00 [2.00, 48.00]	
**2**	6387 (27.93)	6.00 [2.00, 24.00]	
**3**	5941 (25.98)	6.00 [2.00, 24.00]	
**4**	8083 (35.35)	9.00 [2.00, 36.00]	

## Discussion

Tinnitus is considered to be a disease involving different types and degrees of changes in the auditory pathway and auditory nervous system, and previous studies have shown that there are great individual differences in tinnitus perception (including tinnitus frequency, loudness, position, and severity) [24–27]. Based on the individual needs of the patient, the implementation of tailored therapeutic measures may be useful to improve the effects of different treatments [24, 28]. Tinnitus management apps based on smart mobile devices have provided the opportunity for big-data analysis of tinnitus, thus helping clinicians and researchers better understand tinnitus, particularly in identifying symptom severity and tinnitus characteristics in different patients. This aids in individualizing the treatment of tinnitus and in improving the treatment effect. In addition, tinnitus management applications significantly reduce healthcare costs and remove the limitations of traditional medical outpatient treatments regarding treatment times, locations, and numbers of visits.

The FTRS app has been developed since 2015. It is conservatively estimated that there are more than 130 million tinnitus patients in China. We realize that smartphones are ideal for delivering such interventions because they are widely used by adults in China. In the preliminary clinical application, we found that the tinnitus treatment sound generated by using the patented modulation strategy had a good tinnitus-relieving effect. In order to better benefit the majority of tinnitus patients, our team further developed this professional tinnitus diagnosis and treatment app, FTRS, based on our independent patents. It only takes about 10 min to match the type, frequency, and loudness of tinnitus (fast pitch matching and refined pitch matching test). From there the app can customize a personalized tinnitus-relieving treatment sound online (real-time modulation by patented technology), track and evaluate the treatment effect (multi-dimensional tinnitus severity assessment questionnaire), provide a doctor-patient communication platform, make online appointments with tinnitus experts, conduct psychological cognitive education, disseminate tinnitus-related popular science knowledge (created by professional otolaryngologists), and help with tinnitus rehabilitation and auditory health. The app has been popular with tinnitus patients since its launch in May 2018, and it has been continuously maintained and updated.

A total of 22,867 tinnitus users of FTRS were included in this study, and the large quantity of tinnitus data has provided support for clinical tinnitus research. The distribution of FTRS users exhibited large regional differences with users mainly concentrated in Zhejiang, Jiangsu, and Shanghai and being sparsest in northwest China. In China, Jiangsu, Zhejiang, Shanghai, Guangdong, and Shandong are all provinces with large populations and economies. Our app was widely used in these regions, which was consistent with the demographics of the provinces. The radiation range of the FTRS application played an important role in the regional distribution of app users, and differences in the geographical distribution of users may also reflect the fact that the app development hospital (Eye and ENT Hospital, Fudan University) is located in Shanghai, where medical services are mainly concentrated in Shanghai and the surrounding areas. Furthermore, the lack of attention to tinnitus due to regional economic differences [29] is also likely to be an important reason contributing to the regional differences in the numbers of users. In addition, research has found that 19% of users who use smart mobile devices to manage tinnitus are introduced to a tinnitus management application by hearing professionals, family members, or persons online [30].

We also analyzed the incidence rate of tinnitus in different age groups. A statistical study of tinnitus incidence in the United Kingdom revealed that the incidence of tinnitus increases steadily with age, peaking at age 50–59 years and then declining [29]. However, our study showed different results. The age of users in this study was mainly concentrated in the 25–44-year age group, and we speculate that the ability to understand, accept, and utilize new technology might be the reason for this difference. Research has shown that among individuals with tinnitus, approximately 75% of those who do not use a tinnitus management application do not do so because of a lack of understanding of the app, while 11.5% do not possess a mobile phone or tablet [30]. Persons in the 25–44-year age group are the main users of smart mobile devices and are proficient at searching for and using tinnitus management resources, whereas older patients may lack the ability to understand and use tinnitus apps. Because of the difference in app acceptance among different age groups, the incidence rate of tinnitus among app users of all age groups does not represent the general tinnitus population.

Currently, the relationship between gender and tinnitus incidence is unclear. Some studies have shown that tinnitus incidence is independent of gender [29, 31, 32]; however, other studies have reported a higher incidence and more severe depressive symptoms in male patients, whereas female patients suffer more often from tinnitus and have more serious psychosomatic symptoms [24, 33, 34]. In our study, there was also a difference in the incidence of tinnitus between the genders. Before the age of 55, there were significantly more male patients with tinnitus than female patients. However, after the age of 55 years the proportion of female patients with tinnitus exceeded that of male patients and subsequently increased with age. This phenomenon has never been proposed or discussed clinically. We hypothesized that the peak proportion of male patients in the 15–44-year age group might be because male patients in this age group are more exposed to lifestyle and work-related noise and thus the proportion and degree of hearing loss is more serious than in female patients [35, 36]. Hearing loss and noise exposure are both susceptibility factors of tinnitus. After 45 years of age, the proportion of female users gradually increases, which may be related to female menopause hormone levels [37–39]. This is especially likely because the function of the auditory system has been proven to rely on sex hormones [40]. Smith and Hoare proposed a physiological mechanism in which changes in the levels of estrogen and progesterone in the circulation change the chemical composition of internal/perilymph transport within the cochlear cavity. This theory affects the electrochemical pulses produced by cochlear hair cells and thus can produce the “sound” associated with tinnitus [39, 41]. However, sleep disorders and emotional instability are common symptoms in patients with tinnitus and in menopausal women, and negative emotions are not only a susceptibility factor but also a consequence of tinnitus and can thus increase the severity of tinnitus and impede patients’ habituation to tinnitus [42]. We conclude that changes in hormone levels and the influence of negative emotions in menopausal female patients increase the risk and severity of tinnitus. Hormone replacement therapy has been investigated for the treatment of tinnitus in menopausal women, but considering the side effects of hormone therapy, it is not recommended for clinical use [39, 43]. In addition, Richter et al. reported that women showed fewer proactive coping strategies than men [24]. Therefore, the difference in the number of male and female FTRS users could be a consequence of the fact that female tinnitus populations do not actively cope with tinnitus as well as males and thus there might not necessarily be a gender difference in the incidence of tinnitus in the general population.

The correlation between gender and tinnitus frequency is not clear. Partyka et al. suggested that gender has no correlation with tinnitus frequency [44], whereas Zhang and Niemann reported that female patients account for a higher proportion of individuals with low-frequency tinnitus [28, 45]. Our findings agreed with those of Zhang and Niemann. As Fig. 4D shows, tinnitus in male patients was more obvious in the high-frequency range, whereas tinnitus in female patients was perceived more often as a low-frequency sound, with the female tinnitus frequency distribution showing a “U”-shaped curve. At present, there is no commonly accepted clinical explanation for this phenomenon. Some researchers have proposed that hearing loss and excessive noise exposure are common causes of tinnitus [46–49], and male patients tend to have a more common history of noise exposure than female patients, and the probability of high-frequency hearing loss and tinnitus is relatively higher in male patients [35, 36]. In addition, Niemann observed that women have more types of tinnitus than men. In this survey, most tinnitus patients had single-frequency tinnitus, and the number of women in the multi-frequency tinnitus group was slightly higher than that of men, which is consistent with the findings of Niemann [28]. We also identified and statistically analyzed the tinnitus position among the users. Users with unilateral tinnitus outnumbered those with bilateral tinnitus, and tinnitus on the left side was slightly more common than that on the right. This is consistent with the findings of Liu [50].

We further noticed that though thousands of patients started to use the FTRS app for tinnitus management, however, after a long- or short-term, some patients stop using the app continuously. In order to understand the relevant situation of users discontinuing the use of FTRS, we carried out a telephone follow-up to understand and record the users’ application experience and to collect suggestions for improvement. We followed up by telephone with 192 patients with tinnitus who were treated with the FTRS app and inquired about their adherence to FTRS. It was found that 44 patients (22.9%) adhered to the app (more than 2 h a day), and 105 patients (54.7%) did not follow the doctor’s guidance for out-of-hospital sound therapy through FTRS. A total of 43 patients (22.4%) tried but failed to adhere to the FTRS app. For the people who did not continue using the FTRS app, the main reasons were as follows: (1) busy work made it impossible to use it; (2) the short-term treatment of tinnitus did not show significant improvement; (3) older patients could not operate the app well; and (4) the relief/disappearance of tinnitus. To maximize the self-help treatment effect of the FTRS app, we considered (1) improving the regulatory performance of APP, detecting patients with poor adherence, and reminding them as soon as possible and (2) a follow-up team composed of doctors or nurses that remind patients to adhere to the FTRS app by telephone and through regular follow ups. In addition, professional treatment guidance and consultation should be provided.

In this study, most users had grade-4 tinnitus, which affected their normal life and work. These findings show that it is necessary and important to strengthen tinnitus management and intervention, particularly for patients with grade-4 tinnitus who need to be identified early because these patients may become depressed or suicidal. Thus, if tinnitus is not identified in time there may be serious consequences for society and individual families. Compared with one-on-one tinnitus outpatient treatment, the FTRS has no time and space constraints. Through the remote diagnosis, treatment, and supervision provided by the FTRS, we can quickly identify patients with severe tinnitus and provide treatment suggestions to avoid serious adverse consequences, which enhances the significance and value of the tinnitus app.

In summary, the app has the advantages of Internet-based medicine, extends the core patented technology to the mobile phone client in an innovative manner, and provides homogeneous services for public welfare, thus saving huge medical expenses for the country and for individuals. Moreover, the big data for tinnitus diagnosis and treatment obtained through the app can further provide reliable data support for tinnitus clinical research. For example, the analysis of tinnitus characteristics, especially the age-gender differences and gender-frequency differences in tinnitus patients shown in this article will provide clues for future related research.

(A) Information from a total of 52,731 users was initially screened from the back-end of the FTRS app, and 29,864 records with incomplete personal and tinnitus-related information were excluded. (B)The FTRS was first launched in both the iOS and Android application markets in May 2018, with basic functions including tinnitus matching, sound therapy, follow-up evaluation, and popular science for tinnitus. In 2019 and 2020, the FTRS app was further upgraded for personalized and convenient tinnitus treatment by adding various auxiliary functions such as online outpatient appointments and expert consultation, the introduction of sleep breathing guidance, and day/night bimodal sound therapy. (C) Screenshots of the diagnosis, treatment, and evaluation in the FTRS app. The user’s name and profile picture in screenshots have been mosaicked.

## Data Availability

The datasets used and/or analyzed during the current study are available from the corresponding author on reasonable request. The way to access Fudan tinnitus RS APP: i. IOS (Apple), Android (Huawei, Xiaomi, Redmi, VIVO, OPPO, One Plus and other application markets) can directly search “复旦耳鸣RS (Fudan tinnitus RS)” to download and install; ii. Other mobile phone users can scan the code below or search “复旦耳鸣RS (Fudan tinnitus RS)” wechat official account/mini program, download and install after following; iii. Fudan Tinnitus RS old users can update directly, or uninstall the old version, download, and install the new version through the above channels or directly via: https://obs-erming.fduentigo.com/open/h5/open.html.
